# No evidence of SARS‐CoV‐2 transmission through transfusion of human blood products: A systematic review

**DOI:** 10.1002/jha2.263

**Published:** 2021-07-19

**Authors:** William Frank Mawalla, Belinda J. Njiro, George M. Bwire, Ahlam Nasser, Bruno Sunguya

**Affiliations:** ^1^ School of Medicine Muhimbili University of Health and Allied Sciences Dar es Salaam Tanzania; ^2^ School of Pharmacy Muhimbili University of Health and Allied Sciences Dar es Salaam Tanzania; ^3^ School of Public Health and Social Services Muhimbili University of Health and Allied Sciences Dar es Salaam Tanzania

**Keywords:** blood, blood products, bone marrow, covid‐19, haematopoietic stem cells, SARS‐CoV‐2, transfusion, transmission

## Abstract

The presence of viral nucleic material in the circulation poses a theoretical risk of transmission through transfusion. However, little is known about the possibility of the actual transmission through transfusion or transplantation of blood products. A PROSPERO registered systematic review pooled evidence from PubMed/MEDLINE, Google Scholar and CINAHL. The search included studies on severe acute respiratory syndrome coronavirus 2 (SARS‐CoV‐2) transmission through human blood products. In total 537 studies were extracted, and only eight articles (1.5%) were eligible for the final analysis. A total of 14 patients received blood products from coronavirus disease‐2019 (COVID‐19) virus‐positive donors, and six (42.9%) tested negative for COVID‐19 RT‐PCR for up to 14 days post‐transfusion/transplantation. There were no documented clinical details on the COVID‐19 test for eight (57.1%) blood products recipients. Of the eight patients, none of them developed any COVID‐19‐related symptoms. In conclusion, there is limited evidence of transfusion transmission of SARS‐CoV‐2 via human blood products. Consolidation of further evidence, as it emerges, is warranted.

## 1 INTRODUCTION

Blood and blood products transfusion are life‐saving interventions [1]. Advanced therapies such as haematopoietic stem cell (HSC) transplantation are also, in most cases the only chance of cure and/or last resort treatment for some disease conditions [2]. Product safety is of utmost significance in transfusion and HSC transplantation and is held with high regard in transfusion and transplant medicine [3]. The severe acute respiratory syndrome coronavirus 2 (SARS‐CoV‐2) pandemic has threatened the safety and the balance of the existing demand and supply systems [4].

Early reports have found the presence of SAR‐CoV‐2 viral RNA in plasma and lymphocytes of up to a third of patients with COVID‐19 [5, 6, 7, 8, 9, 10]. The presence of viral nucleic material in the circulation poses a theoretical risk of transmission through transfusion [11]. However, little is known about the possibility of the actual transmission of the virus [12, 13]. To date, isolation of an intact infectious virus is yet to be reported. This has left most blood donation agencies' recommendations on SARS‐CoV‐2 and blood product transfusion to largely draw from limited evidence of past similar respiratory virus outbreaks [14, 15, 16]. This review seeks to gather current evidence on the transmission of SARS‐CoV‐2 via blood and its derivatives.

## 2 MATERIALS AND METHODS

### 2.1 Design

A systematic approach was engaged in searching the literature to try to answer a research question: 'What is the likelihood of SARS‐CoV‐2 transmission through human blood products?'. The review protocol was developed as per Preferred Reporting Items for Systematic Reviews and Meta‐Analyses Protocols guidelines. The protocol is registered in the International Prospective Register of Systematic Reviews (https://www.crd.york.ac.uk/prospero/: PROSPERO database registration number: CRD42020223479).

### 2.2 Search strategy

A systematic evidence search was done using three strategies. First, a team of independent reviewers conducted a search through three major medical databases namely PubMed/MEDLINE, Google Scholar and CINAHL. Second, the team searched for evidence through the websites of key healthcare organizations such as the World Health Organization, Centre for Disease Control and Prevention. Third, a grey literature search was done with help of Google and evidence from reference list of gathered evidence. Evidence search was limited to publications from December 31, 2019, a date where SARS‐CoV‐2 (COVID‐19) was reported from Wuhan, China, to April 2021, evidence published in English and research conducted in human beings. A search strategy was formulated by an experienced librarian for PubMed/MEDLINE (Additional file 1) and adjusted for other search databases. A wide range of inclusivity around the theme of blood and/or blood products transfusion, haematopoietic stem cell transplantation and SARS‐CoV‐2 transmission was aimed. Key terms included 'blood transfusion', 'blood products', 'haematopoietic stem cell transplantation', 'transfusion', 'transmission', 'blood', 'Severe Acute Respiratory Syndrome Coronavirus‐2', 'SARS‐CoV‐2' and 'Coronavirus Disease 2019', 'COVID‐19'.

### 2.3 Eligibility criteria and study selection

The inclusion criteria were based on studies describing blood or HSC donors with confirmed SARS‐CoV‐2 and recipients of blood or HSC from donors with confirmed SARS‐CoV‐2 within 14 days of donation. The duration was chosen owing to the average maximum time it takes for the symptomatic presentation of COVID‐19 [17]. Recipients of blood or HSC with known exposure to cases or suspects of SARS‐CoV‐2 and/or tested positive for SARS‐CoV‐2 before transfusion were excluded. Study selection was managed using Covidence software (Australia) where two independent reviewers (WFM and BJN) evaluated articles for potential inclusion by screening titles and abstracts followed by assessment of full‐text articles to determine eligibility for final inclusion. Between each assessment, results were discussed to reach a consensus on the interpretation of inclusion criteria. Based on the availability of literature, the review included letter to the editor, correspondence, editorial and research articles including case reports. Reviews and studies whose data were obtained from animal and in‐vitro studies were excluded.

### 2.4 Data management

**FIGURE 1:**
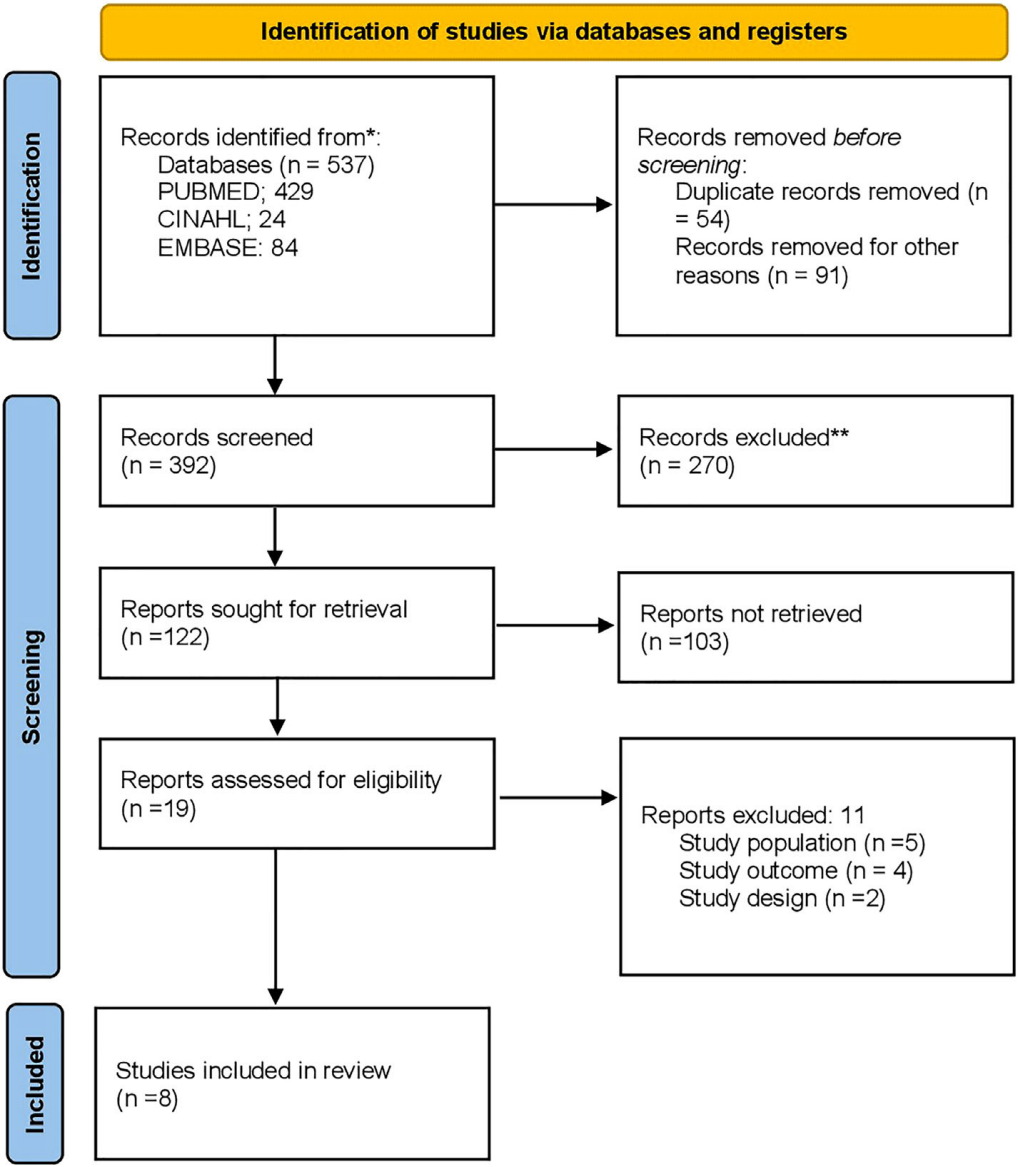
PRISMA diagram showing the flow of article search and screening

Duplicate publications were identified and removed using Covidence software (Australia). Identified publications were analyzed using criteria based on blood or HSC donors with confirmed SARS‐CoV‐2 and recipients of blood or HSC from donors with confirmed SARS‐CoV‐2 and maximum correspondence with inclusion criteria and minimal risk of bias (Figure 1).

### 2.5 Data extraction

Data were extracted from eligible studies and transferred to an Excel spreadsheet 2010 (Microsoft Corporation) form (Table 1). The primary outcome was recipients of blood products and/or HSC from donors with confirmed SARS‐CoV‐2 who contract SARS‐CoV‐2 as a result of transfusion or transplantation. COVID 19 is a relatively new disease; therefore, all extracted studies were expected to be case series or case reports, and with small participant numbers per each study. This led the authors not to perform tests for heterogeneity or meta‐analysis.

**TABLE 1:** Characteristics of the included studies

Author	Country	Design	Type of product	Type of donor test	Presence of antibodies in donors	Donor testing post‐donation (days)	Transfusion/Transplantation post‐donation(days)	COVID‐19(positive recipients/total)	Presence of covid‐related symptoms (positive recipients/total)	Risk assessment (NOS); 0: high, 10: low
Cappy et al.	France	CR	Platelets*	RT‐PCR (Plasma)	Negative	4	3	NA	0/1.0	6
P. Lázaro del Campo, et al.	Spain	CR	Mobilized peripheral blood CD34+ cells	RT‐PCR (Nose Swab)	NR	3	NR	0/1.0	0/1.0	9.25
Al‐Essa et al.	Saudi Arabia	CR	HSC**	RT‐PCR (Nose Swab)	NR	6	4	0/1.0	NR	7.5
Anurathapan et al.	Thailand	CR	Bone marrow stem Cells	RT‐PCR (Nose Swab)	NR	0	0	0/1.0	0/1.0	9
Luzzi et al.	Brazil	CR	Platelets	RT‐PCR (Nose Swab)	NR	5	5	NA	0/1.0	5.4
		CR	Platelets	RT‐PCR (Nose Swab)	NR	5	4	NA	0/1.0	
		CR	Platelets	RT‐PCR (Nose Swab)	NR	5	4	NA	0/1.0	
		CR	Whole blood	RT‐PCR (Nose Swab)	NR	1	4	NA	0/1.0	
		CR	Granulocytes	Serology***	NA	14	0	NA	0/1.0	
		CR	Platelets	Serology***	NA	14	2	NA	0/1.0	
Cho et al.	Republic of Korea	CR	Platelets	RT‐PCR****		3	2	0/1.0	0/1.0	7.2
Maakaron et al.	USA	CR	Bone marrow stem cells	RT‐PCR****	NR	2	NR	NA	0/1.0	5.5
Leclerc et al.	France	CR	Mobilized peripheral blood CD34+ cells	RT‐PCR (Nose Swab)	NR	0	0	0/1.0	0/1.0	8
		CR	Mobilized peripheral blood CD34+ cells	RT‐PCR (nose swab)	NR	0	0	0/1.0	0/1.0	

Abbreviations: CD, cluster of differentiation; COVID‐19, coronavirus disease 2019; CR, case Report; HSC, haematopoietic stem cells; NA, not applicable; NOS, Newcastle – Ottawa Scale; NR, not recorded; RT‐PCR, reverse transcription polymerase chain reaction.

* Pathogen‐reduced platelets.

** Not specified whether mobilized peripheral blood HSC or bone marrow HSC.

*** Not specified whether antigen or antibody testing.

**** Type of specimen not specified

### 2.6 Quality assessment

The quality of the included studies (risk of bias) was assessed using the Newcastle Ottawa Scale adapted for cross‐sectional studies (additional file S2) as described elsewhere [18]. The tool was customized to assess studies on (SARS‐CoV‐2) transmission through human blood products. The risk of bias was evaluated by two independent reviewers (WFM and BJN). Discrepancies were resolved by consensus, and/or consulting a third reviewer (GMB) where necessary

### 2.7 Summary measures and synthesis of results

A summary of the proportion of COVID‐19 positive blood and/or HSC recipients among all blood and/or HSC recipients from COVID‐19 positive donors was documented as obtained from the main article. The narrative was written by the lead reviewer (WFM) and reviewed independently by two reviewers (BJN and GMB). Missing variables from included studies were documented as not reported (NR).

## 3 RESULTS

### 3.1 Characteristics of the included studies

A systematic search extracted a total of 537 studies, and only eight articles (1.5%) [18, 19, 20, 21, 22, 23, 24, 25] were eligible for the final analysis. Of the eight studies included, two (25%) were case series [18, 25], and six (75%) were case reports [19, 20, 21, 22, 23, 24]. Across all studies, a total of 14 individuals received blood products from COVID‐19 virus‐positive donors. All studies reported on the possibility of COVID‐19 transmission to recipients in the post‐transfusion/transplantation period through recipient COVID‐testing and/or assessment of COVID‐related symptoms (Table 1).

More than a third of the studies [19, 20, 25] were conducted in Europe (France and Spain). Platelets and HSC were the most common transfused/transplanted blood products, transfused or transplanted to six (42.8%) recipients, each. RT‐PCR was used to test most donors, 12 (85.7%). One study [18] reported serology testing was done in two (14.3%) donors without specifying whether the testing was for antigen or antibody. The clinical specimen used was a nose swab in nine (64.3%) [18, 20, 21, 22, 25], plasma in three (21.4%) [18, 19], and in two (14.3%) donors [23, 24] the specimen used was not specified. Donor testing and product transfusion or transplantation post‐donation were within 72 h in 50% of all the cases. Two studies [20, 24] did not report the time of product transplantation post‐donation (Table 1).

### 3.2 Suspected transfusion transmission of SARS‐CoV‐2

A total of 14 patients received blood products from COVID‐19 virus‐positive donors, six (42.9%) tested negative for COVID‐19 RT‐PCR for up to 14 days post‐transfusion/transplantation. For the other eight (57.1%) recipients, details of the COVID‐test were not reported; however, none of these recipients are documented to have developed any COVID‐related symptoms (Table 1).

Of the six patients who tested negative, patient number one [20] was a 57‐year‐old male who had relapsed mantle cell lymphoma and underwent a matched sibling Haematopoietic Stem Cell Transplantation (HSCT) in Spain. The HSC donor was her sister, who had mobilized peripheral blood HSC collected through apheresis. The transplantation went on uneventful, and reports of the donor testing positive for COVID‐19 (nose swab) came 72 h after the transplantation. The recipient COVID‐19 testing was done on the day of the report and continued after every 48 h for 24 days. All tests came out negative for COVID‐19 RT‐PCR. Three months later, the patient had attained complete remission and had not developed any COVID‐related symptoms or antibodies (both IgM and IgG).

Patient number two [21] was a 2‐year‐old boy who developed a need for platelet transfusion 22 days after undergoing a matching‐sibling HSCT for the treatment of acute leukaemia (pre‐B‐ lymphoblastic leukaemia). He was transfused with a single donor apheresis platelet unit from a donor who tested positive for COVID‐19 (nose swab) 5 days after donating. The patient was followed up with serial COVID‐19 testing (blood and nose swab for RT‐PCR) for up to 14 days and tested negative without any COVID‐19‐related symptoms.

Patient number three [22] was a 7‐year‐old female who underwent a matched‐sibling HSCT for treatment of beta‐thalassemia‐haemoglobin E. The donor was her younger brother who tested positive for COVID‐19 (nose swab) on the day of HSC harvesting. Nevertheless, the transplant went ahead, and the patient was followed up with serial blood and nose swab RT‐PCR and antibody tests for up to 14 days post‐transplantation. All tests came out negative, and the patient never developed antibodies or any immediate transplant‐related complications.

Patient number four [23], a 21‐year‐old Korean male who presented with severe aplastic anaemia on supportive transfusion therapy was transfused with platelets from a donor who tested positive for COVID‐19 (nose swab) 3 days post‐donation. The patient was followed up for 14 days and did not develop any COVID‐19‐related symptoms and tested negative with serial RT‐PCR tests. Patients five and six [25] were 60‐year‐old males with acute myeloid leukaemia who underwent HSCT from two different donors who both tested positive for COVID‐19 (nose swab) on the day of HSC collection. Both patients were followed up for a month and remained negative for COVID‐19 (plasma and nose swab for RT‐ PCR) without any COVID‐related symptoms.

## 4 DISCUSSION

To the best of our knowledge, this is the first systematic review documenting the evidence of transmission of COVID‐19 through transfusion or transplantation of blood products. None of the recipients of blood products from donors who were confirmed to have COVID‐19 tested positive or developed COVID‐19‐related symptoms post‐transfusion or transplantation.

The results further strengthen the findings from a few small studies that have reported no evidence of transmission of respiratory virus, in particular, the Middle East Respiratory Syndrome‐CoV and SARS‐CoV through blood product transfusion [11, 18, 26]. This is despite the demonstrated presence of viral RNA in the blood of infected individuals [8, 9, 27].

The current review included studies describing various types of donated blood products, including whole blood, platelets, granulocytes and mobilized peripheral blood stem cells from COVID‐19 positive donors. One study [19] reported the blood component (platelet) from the COVID‐19 positive donor was pathogen‐reduced before being transfused to the recipient; however, the technique was not explained nor the effectiveness of the technique against SARS‐CoV elaborated. Whether the currently employed post‐donation processing of blood components and stem cells and pathogen reduction methods can deactivate SAR‐CoV and prevent transmission is yet to be determined.

Furthermore, half of the studies included in the current review used a nasal swab to test donors for COVID‐19 infection [18, 21, 22, 25]. One study [19] tested plasma, and two [23, 24] used RT‐PCR for testing; however, the latter did not report the type of specimen used. Testing the presence of the virus antigen in the blood product being transfused or transplanted would give a more precise measurement since the shedding of the virus from the primary site (respiratory tract) to the circulation occurs at a later stage of infection. In previous studies on coronavirus, SARS‐CoV RNA was detected in serum during the first week after the onset of symptoms in nearly 80% of patients and dropped to 50% in the second week [6, 28, 29]. The role of the presence and precise time of detection of the virus antigen in the circulation is therefore still of unknown significance in the transmission of the virus [8]

The small number of studies and participants obtained and the type of study designs (low‐quality study designs, i.e., retrospective in nature) limit the conclusions of the current review. Nevertheless, being the first review that gathers evidence of COVID‐19 transmission in blood products, the findings are a key in building evidence that examines the possibility of transmission of SARS‐CoV‐2 through blood products. This is particularly useful in the development of donor deferral criteria for blood donation agencies and combating the shortage and wastage of blood products, which are already limited in most regions.

## 5 CONCLUSION

There is limited evidence of transfusion transmission of SARS‐CoV‐2 via human blood products. Consolidation of further evidence on the possibility of transfusion transmission from new studies, as they appear, is warranted.

## CONFLICT OF INTEREST

The authors declare that they have no competing interests.

## AUTHORS CONTRIBUTIONS

William Frank Mawalla and Belinda J. Njiro designed the study protocol, conducted data extraction and synthesis. William Frank Mawalla drafted the narrative synthesis. George M. Bwire participated in revising the protocol, revising the narrative synthesis and manuscript. Ahlam Nasser performed a data search and revised the manuscript. Bruno F. Sunguya participated in protocol development and revised the narrative synthesis. All authors have read and approved the final version of this manuscript

## Supporting information

SUPPORTING INFORMATION

SUPPORTING INFORMATION

## Data Availability

All data sets used and/or analyzed in this review are provided in the main manuscript and its supplementary materials (Additional file 1 and 2).
