# Supplementary material for: No evidence of SARS‐CoV‐2 transmission through transfusion of human blood products: A systematic review
Source: EJHaem. 2021 Jul 19;2(3):601–6. doi: 10.1002/jha2.263 (PMC8426699; doi:10.1002/jha2.263)
Supplement: Supplementary file 2 — SUPPORTING INFORMATION [file JHA2-2-601-s001.docx]

# No evidence of SARS-CoV-2 transmission through transfusion of human blood products: a rapid systematic review

*William Mawalla, Belinda Njiro, George Bwire, Ahlam Nasser, Bruno Sunguya*

**Additional file 2:** Assessment of risk of bias using Newcastle-Ottawa Scale adapted for cross-sectional studies

| **Study** | **Quality parameter** | **Maximum stars** | **Score** | **Reason (s)** |
| --- | --- | --- | --- | --- |
| Cappy etal. (18) | Selection | 6 | 5 | Donor testing took place 72 hours following blood product donation. |
|  | Outcome | 4 | 1 | Even though the recipient remained symptomless, recipient testing post-transfusion was not done. |
|  | **Total** | **10** | **6** | **Moderate bias** |
| P. Lázaro del Campo, et al. (19) | Selection | 6 | 5.5 | The donor was tested using RT-PCR and testing was done within 72 hours of donation. |
|  | Outcome | 4 | 3.75 | The recipient had his first RT-PCR test after 72hours and had follow-up tests done. A description of whether pathogen reduction techniques to the donated product before transplantation were employed or not, is not made. |
|  | **Total** | **10** | **9.25** | **Low bias** |
| Al-Essa et al. (23) | Selection | 6 | 4.5 | The donor was tested using RT-PCR, however, was done after 72 hours of donation. |
|  | Outcome | 4 | 3 | The recipient had his first RT-PCR test after 72hours and had follow-up tests done. |
|  | **Total** | **10** | **7.5** | **Low bias** |
| Anurathapan et al. (24) | Selection | 6 | 5.5 | The donor was tested using RT-PCR and testing was done within 72 hours of donation. |
|  | Outcome | 4 | 3.5 | Even though the recipient had his first RT-PCR test (from blood and nasopharyngeal specimen) after 72hours; he had follow-up tests done until 14 days post-transplant. |
|  | **Total** | **10** | **9** | **Low bias** |
| Luzzi etal. (17) | Selection | 6 | 4.4 | Most donors were tested using RT-PCR, however, in most, it was done after 72 hours of donation |
|  | Outcome | 4 | 1 | Even though the recipient remained symptomless, recipient testing post-transfusion was not done. A description of whether pathogen reduction techniques to the donated product before transplantation were employed or not, is not made. |
|  | **Total** | **10** | **5.4** | **Moderate bias** |
| Cho etal. (26) | Selection | 6 | 4.5 | The type of clinical specimen used in testing donor positivity is not described. |
|  | Outcome | 4 | 2.75 | The type of clinical specimen taken to test the recipient is not described. A description of whether pathogen reduction techniques to the donated product before transplantation were employed or not, is not made. |
|  | **Total** | **10** | **7.25** | **Moderate bias** |
| Maakaron etal. (25) | Selection | 6 | 4.5 | Donor testing took place 72 hours following blood product donation; however, the type of clinical specimen used in testing donor positivity was not described. |
|  | Outcome | 4 | 1 | Even though the recipient remained symptomless, recipient testing post-transfusion was not done. A description of whether pathogen reduction techniques to the donated product before transplantation were employed or not, is not made. |
|  | **Total** | **10** | **5.5** | **Moderate bias** |
| Leclerc et al. (20) | Selection | 6 | 5.5 | The donor was tested using RT-PCR and testing was done within 72 hours of donation. |
|  | Outcome | 4 | 2.5 | The recipient testing was done using RT-PCR (Plasma and nasopharyngeal) however the timing of the first testing is not indicated. A description of whether pathogen reduction techniques to the donated product before transplantation were employed or not, is not made. |
|  | **Total** | **10** | **8** | **Low bias** |

**Interpretations**

1. In this scale, the selection was mainly assessed based on a) the timing of donor testing after blood product donation, b) the type of test used to ascertain donor positivity and c) the type of clinical specimen used in testing donor positivity.
2. The outcome was mainly assessed based on a) the recipient first test and follow-up testing after blood product donation, b) the type of test used for the Recipient(s), c) the type of clinical specimen used and d) the pathogen reduction measures to the blood products before transfusion/transplantation.
3. Since all studies were case reports and case series, the comparability was not assessed.

**Acknowledgement**

This tool was adapted from the study by Bwire, GM, Majigo, MV, Njiro, BJ, Mawazo, A. Detection profile of SARS-CoV-2 using RT-PCR in different types of clinical specimens: A systematic review and meta-analysis. *J Med Virol*. 2021; 93: 719– 725. <https://doi.org/10.1002/jmv.26349>
